# Assessing feasibility of conducting medication review with follow-up among older adults at community pharmacy: a pilot randomised controlled trial

**DOI:** 10.1007/s11096-024-01711-3

**Published:** 2024-04-18

**Authors:** Christina Malini Christopher, Ali Qais Blebil, KC Bhuvan, Deepa Alex, Mohamed Izham Mohamed Ibrahim, Norhasimah Ismail, Mark Wing Loong Cheong

**Affiliations:** 1 School of Pharmacy, Monash University Malaysia, Jalan Lagoon Selatan, Subang Jaya, Selangor Malaysia; 2 School of Clinical Sciences, Queensland University of Technology, Brisbane, Australia; 3 Jeffrey Cheah School of Medicine and Health Sciences, Monash University Malaysia, Jalan Lagoon Selatan, Subang Jaya, Selangor Malaysia; 4 Clinical Pharmacy and Practice Department, College of Pharmacy, QU Health, Qatar University, Doha, Qatar; 5 Bayan Lepas Health Clinic, Ministry of Health, Bayan Lepas, Penang Malaysia; 6 College of Public Health, Medical and Veterinary Sciences, James Cook University, Townsville, QLD Australia; 7 Department of Geriatrics and Healthy Living, KIMSHEALTH, Thiruvananthapuram, Kerala India

**Keywords:** Community pharmacy, Feasibility, Medication review, Older adults, Pilot RCT

## Abstract

**Background:**

Medication review with follow-up is essential for optimising medication utilisation among the older adult population in primary healthcare.

**Aim:**

This study aimed to evaluate the feasibility of implementing medication reviews with follow-up for older adults in community pharmacies and examined potential outcomes on medication use.

**Method:**

A pilot randomised controlled trial was conducted with 4 cluster-randomised community pharmacies to assess the feasibility of the intervention. Two community pharmacies served as intervention and control groups. Both groups recruited older adults over 60 who were followed over 6 months. The translated Medication use Questionnaire (MedUseQ) was administered at baseline and 6 months for both groups. The outcomes were to assess the feasibility of conducting medication review with follow-up and the probable medication use outcomes from the intervention.

**Results:**

The intervention and control groups comprised 14 and 13 older adults. A total of 35 recommendations were made by pharmacists in the intervention group and 8 in the control group. MedUseQ was easily administered, providing some evidence the feasibility of the intervention. However, there were feasibility challenges such as a lack of pharmacists, collaborative practice, difficulties with the tool language, time constraints, and limited funds. Questionnaire results provided a signal of improvement in medication administration, adherence, and polypharmacy among intervention participants. The incidence of drug related problems was significantly higher in the control group (median = 1) after 6 months,* U* = 15, *z* = − 2.98, *p* = 0.01.

**Conclusion:**

Medication review with follow-up is potentialy practical in community pharmacies, but there are feasibility issues. While these challenges can be addressed, it is essential to study larger sample sizes to establish more robust evidence regarding outcomes.

*Clinical trial registry*: ClinicalTrials.Gov NCT05297461.

**Supplementary Information:**

The online version contains supplementary material available at 10.1007/s11096-024-01711-3.

## Impact statements

- The study emphasized the feasibility issues of conducting medication review with follow-up at community pharmacies, focusing on recruitment, retention, questionnaire administration and time costs.
- The findings highlighted a signal of improvement in medication use outcomes favouring a community pharmacist-led intervention.
- This feasibility study would be useful for policymakers to potentially implement strategies to enhance effective medication utilization.

## Introduction

Advances in healthcare have resulted in an increase in life expectancy, leading to a higher prevalence of individuals with multiple health problems consuming multiple medications [1]. This increased medication use among older adults has contributed to medication-related problems, such as polypharmacy, adverse drug incidences, inappropriate medication use, and a need to deprescribe potentially inappropriate medicines [2]. Inappropriate medication use can result in adverse drug events, poor treatment outcomes, treatment failure and increased morbidity and mortality, including hospitalisation [3]. This significantly increases healthcare utilisation, thereby draining resources from the healthcare system and affecting the delivery of other health system programs and priorities [4].

Primary healthcare providers, particularly community pharmacists, are frequently the first point of contact for older adults seeking healthcare services in several countries including Malaysia [5, 6]. Extended interventions involving cognitive pharmaceutical services such as medicine reviews, screening and monitoring health outcomes, and treatment adherence programs are currently offered at community pharmacies [7]. In addition, interventions such as medication review, providing education, pharmaceutical care plans, counselling, and electronic reminder devices are being conducted globally [8–11]. In recent years, several publications have appeared documenting post-discharge medication review as part of community pharmacist intervention for the ageing population [12–14]. In addition, several studies provided evidence that community pharmacists substantially reduce falls, adverse drug events, hospitalisations and improve medication adherence through medication review among older adults [15–18].

In primary care settings, community pharmacists are crucial in improving medication use among older adults and reducing their health burden. Medication review intervention is a clinical process where the pharmacist reviews the patient’s medication, identifies drug-related problems, and suggests strategies to reduce medication use problems. Such interventions have been utilised in various countries, particularly in Organisation for Economic Co-operation and Development (OECD) regions, including Australia, New Zealand, and the United Kingdom, with pharmacists being remunerated for their services [7]. However, the scope for intervention remains limited in low- and middle-income countries (LMICs) because of a lack of recognition of pharmacist expertise and poor pharmacy structure and support system at the primary care level.

In Malaysia, the primary healthcare system focuses more on public health clinics as these healthcare services are funded by the government [19]. Most older adults visit primary healthcare (PHC) facilities including PHC clinics and centers, and community pharmacies (private) [20]. Hamidi et al. [21] identified that about 10.3% of the general population visited community pharmacies for health purposes in Malaysia in 2021, compared to 5% in 2015 [22]. Community pharmacists in Malaysia are mostly involved in dispensing and supplying medicines and supplements, suggesting treatment for minor illnesses and conducting health screening tests [23]. Besides, community pharmacies also serve as an alternative healthcare facility for public seeking care for minor health issues as the public health clinics are congested and overcrowded [24]. Community pharmacies in Malaysia are privately run and do not provide any publicly funded health services and medicine. Unfortunately, there has been under-utilisation of community pharmacist services in Malaysia [25, 26]. Medication reviews are not commonly provided to older adults at most community pharmacies in Malaysia, primarily because this service has yet to be incorporated into the government healthcare system. As a result, it is not funded through public service channels [27]. Moreover, dispensing separation has contributed to fewer older adults being reviewed for their medications in community pharmacy settings [28]. For instance, medication reviews have been proposed under the Community Pharmacy Benchmark Guidelines; however, there is a lack of utilisation of medication reviews focusing on older adults [29]. A recent study conducted in Malaysia showed that medication review improved medication adherence and knowledge and reduced drug-related problems [30]. Similarly, studies have emphasised the improvement in clinical outcomes among older adults [31, 32]. In addition, Karuppannan et al. [33] concluded that it is feasible to conduct medication reviews in community pharmacies in Malaysia and identify drug-related problems such as adverse drug events. However, the study only focused on the issue from the perspective of community pharmacists, but the feasibility was not tested in practice. Therefore, there is a need to focus on feasibility aspects and contribute to effective medication utilisation among older adults.

## Aim

This pilot randomised controlled trial aimed to assess the feasibility of implementing a medication review with follow-up to evaluate medication use outcomes among older adults in northern Malaysia.

### Ethics approval

This study followed the declaration of Helsinki and the Consort Standards of Reporting Trials [34]. The trial was registered in ClinicalTrials.gov NCT05297461. Ethical approval was obtained from the Monash Human Research Ethical Committee (31954) on 7th March 2022.

## Method

### Study sites

A letter inviting community pharmacies in Penang state was initiated through the Malaysian Pharmaceutical Society. There are a total of 229 community pharmacies with a ratio of pharmacists 1:7749 in Penang [35]. Six community pharmacies expressed their interest in participating in this study. After a briefing on the study objectives and potential participants, four of the community pharmacies remained interested and were recruited. Four independent community pharmacists with an average working experience of 10 years were recruited. We included only independent community pharmacies to avoid any bias.

### Randomisation

A pilot RCT using a two-arm parallel model with 1:1 allocation was used. A cluster-randomised design was adopted to identify cluster intervention and control groups. A restricted randomisation technique was applied to match the paired community pharmacies to be similar in terms of the number of staff (high number of staff or some staff), type of participant visitation (older or younger patients), and location(urban or rural) [36]. Thus, two community pharmacies served as the intervention groups and two as control.

### Eligibility criteria

**Fig. 1:**
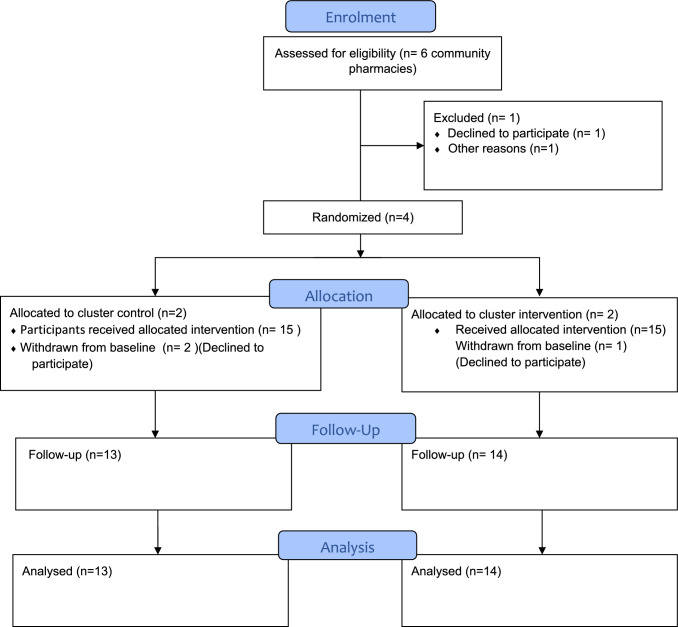
CONSORT Flow chart

Target participants were recruited by participating community pharmacies between April 2022 and May 2022 if (i) Aged 60 and above, (ii) Had been patronising the pharmacy for purchasing a minimum of one prescription medication for at least the past 3 months, (iii) Could converse, read and understand the Malay language or English language and (iv) Had access to a telephone or the internet (Fig. 1). Prior to the start of data collection, informed consent was obtained.

### Sample size

There was no formal calculation for the sample size, but given the importance of size justification [37], the rule of thumb was applied to have 30 participants and at least 12 participants in each arm [38]. From previous feasibility studies which explored pharmacist-facilitated medicines review interventions, a size of 30 participants was selected [39, 40].

### Description of intervention

Medication review with follow-up was the intervention conducted. Community pharmacy benchmarking guideline Pharmaceutical Services Division provides medication review guidelines in Malaysia’s community pharmacy settings [29] (Supplementary material A). The protocol consists of reviewing medications, identifying inappropriate and adverse drug events, educating the indication, dose frequency of medications and compliance, and proposing a plan of action for each patient. Medication review intervention by community pharmacists serves as an essential component of patient care, promoting safe and effective medication use and optimising patient health. Currently, medication review service is optional in community pharmacies. During the medication review, the pharmacist advises each medication indication and safety profile, reviews any complementary or traditional medications, and ensures appropriate medication administration. In addition, participants are educated on adverse drug events and the importance of medication adherence. Pharmacists identify inappropriate medication, doses, and frequencies and discuss with prescribers how to optimize the treatment plan. Finally, follow-up was conducted at the second, fourth, and end of the study to ensure appropriate medication and to identify any drug-related problems. Follow-up included detecting any drug-related problems, updating current medication regimens and clarifying any further queries. The follow-up was conducted either physically or via telephone.

### Intervention group

Before intervention initiation, all the community pharmacists in the intervention pharmacies underwent half-day training on the intervention and the study processes. Older adult participants were recruited based on the eligibility criteria. Participants received the intervention at 0 months and followed-up at the second and fourth months, and end of study. In addition, participants were given a cross-cultural adapted Medication Use (MedUseQ) questionnaire at baseline and end of the study [41]. This questionnaire was used to identify any medication use problems (Supplementary material B).

### Control group

Control groups were given an adapted MedUseQ questionnaire to be administered at baseline and 6 months. Standard care included the usual medication dispensing and counselling. Pharmacists in the control group were briefed only on the study processes.

### Outcomes

The primary outcome centred on assessing the feasibility of the research process on (a) Recruitment, retention, and time; (b) Pharmacist recommendations to prescribers; (c) Frequency of medication administration problems(e.g. difficulties in pressing canisters, cutting tablets), medication adherence, accessibility, polypharmacy, knowledge score (understanding the indication, dose, frequency and side effects of own medications), and (d) Drug-related problems such as adverse drug events, number of medications, and medication issues detected, i.e. frequency, dose, duration of medications [42]. The outcomes of the research process were obtained through seeking verbal feedback from pharmacists on the experiences of providing the intervention to older adult participants.

### Data collection

Demographic data were collected from June 2023 till November 2023 for both intervention and control groups at baseline. A self-administered questionnaire was adapted, translated and modified from the Medication Use Questionnaire (MedUseQ) to assess medication use among older adults in Penang [41]. Medication review interventions were performed for the intervention group, while standard care was provided for the control group. The intervention group received medication review intervention with follow-up by trained community pharmacists, including reviewing older adults’ medications and identifying drug-related problems. Community pharmacists reviewed the medications and tried to resolve any drug-related problems encountered by the participants. In addition, the pharmacists conducted a question–answer session with the participants to enhance older adults’ knowledge and awareness of their medications. Intervention groups were followed up at 2 months and 4 months through a phone call or face-to-face. Both group participants were followed up 6 months after the intervention and given the MedUseQ survey to compare baseline and end of study. Verbal feedback on feasibility issues from pharmacists was noted.

### Data analysis

Data analysis was performed using the IBM Statistical Package for Social Sciences (IBM SPSS), version 26.0 (Armonk, NY: IBM Corp). Descriptive analysis of demographics, intervention group medications and prescriber recommendations was conducted. Statistical tests for inferential statistics were set at a 95% confidence level. Whilst acknowledging that the pilot randomised study was not powered for any outcomes, Mann–Whitney U Test or Fishers exact test was used to explore the statistical significance between the baseline demographics and Mann–Whitney U test to assess the differencse in the drug-related outcomes between intervention and control groups at 6 months. Intention-to-treat (ITT) analysis was utilised, maintaining participants’ original randomisation assignments.

## Results

A total of 30 older adults was recruited; 15 were each allocated to intervention and control groups based on their randomised community pharmacies. One participant from the intervention and two from the control group withdrew before the study, declining to participate after being briefed. Thus, 13 participants in the control group and 14 in the intervention group completed the study. The baseline demographics of both groups is shown in Table 1.

**Table 1:** Baseline demographics of intervention and control groups

Demographic variable	Intervention, n (%)	Control, n (%)	*p*-value*
*Gender*			0.59
Male	8(57.1)	5(38.5)	
Female	6(42.9)	8(61.5)	
*Age*			0.07**
Mean (Years)	72.7	66.9	
Age Range (Years)	60–86	60–73	
*Ethnicity*			0.04
Malay	3(21.4)	3(23.1)	
Chinese	10(71.4)	8(61.5)	
Indian	1(7.1)	2(15.4)	
*Education level*			1.00
No formal education	0	5(38.5)	
Primary	3 (21.4)	6(46.2)	
Secondary	8 (57.1)	2(15.4)	
Tertiary	3 (21.4)	0	
*Living arrangement*			1.00
Alone	1 (7.1)	6(46.2)	
Aged/Nursing home	1 (7.1)	0	
Family	12 (85.7)	7(53.8)	
*Living income source*			0.46
Working	1 (7.1)	2(15.4)	
Pension	10 (71.4)	1(7.7)	
EPF/Retired fund	1 (7.1)	0	
Social welfare support	0	5(38.5)	
Family support	2(14.3))	5(38.5)	
*Household income*			0.12
B40 (Less than Rm4850)	6 (42.9)	8(61.5)	
M40 (Rm4851-Rm10970)	8 (57.1)	4(30.8)	
T20 (More than Rm10971)	0	1(7.7)	
*Do you have health insurance?*			0.29
Yes	4 (28.6)	7(53.8)	
No	10 (71.4)	6(46.2)	
*Do you spend on healthcare monthly?*			0.07
Yes	3 (21.4)	7(53.8)	
No	11 (78.6)	6(46.2)	
Average	RM 520 (RM250-RM1000)	RM680 (RM64-RM5000)	
*Number of medications*			0.11
1–3	8(57.1)	4(30.8)	
4–6	3 (21.4)	7(53.8)	
7–10	2(14.3)	1(7.7)	
More than 10	1(7.1)	0	

*Fishers’s Exact test

**Mann–Whitney U test

### Outcomes

#### Assessing the feasibility of recruitment and delivery of intervention

Verbal feedback from community pharmacists in the intervention group highlighted a few challenges such as a lack of pharmacists at their premises and time constraints which hindered the medication review follow-up process. One pharmacist mentioned that the follow-up process was challenging as some participants had mobility difficulties and could not be easily reviewed via telephone. Also, lack of communication with general practitioners, especially in private settings contributed to difficulty obtaining patients’ medical records and history, and thus reviewing prescriptions. Control group pharmacists mostly had difficulties administering the questionnaire due to as language barriers. Table 2 gives the feedback from the intervention and control groups on the recruitment and delivery processes of the intervention.

**Table 2:** Feasibility issues reported from intervention and control group pharmacies

Questions	Pharmacy A (Intervention)	Pharmacy B (Intervention)	Pharmacy C (Control)	Pharmacy D (Control)
How long it took to recruit participants	1.5 months	2 months	1 month	1 month
Retention of participants at the end of study	77.8%	77.8%	66.7%	77.8%
Issues of time for providing intervention or administrating the questionnaire	No issue	Yes, roughly 45 min per participants for the intervention	Participants needed more time to answer the questionnaire	No issue
Ease of administering the questionnaire	Easy to understand	Difficult for language barrier participants	Difficult for language barrier participants	Easy to understand
Other challenges in providing the intervention	Busy schedule was a limitation for me. Follow-up was a bit challenging as some participants had mobility issues and thus could not be conducted through phone calls	More time and more staff at the premise would be helpful to administer the intervention without interruption. Difficult to call prescribers to discuss prescription error sometimes	Probably lack of staff, was a little challenging to administer the questionnaire	More challenges in recruiting participants as some refused to join

#### Pharmacists’ recommendations or interventions

Table 3 shows the recommendations or interventions on medication use and disease management for the intervention group participants. A total of 30 recommendations were provided by pharmacists. Eleven suggestions to prescribers centred on revising the prescriptions; all suggestions were accepted. These involved identifying inappropriate medications and reducing the dose and frequency of medications. Six counselling sessions were held; a further 2 recommendations were made on monitoring hypertension and diabetes parameters, and 8 recommendations were implemented on providing patient education on lifestyle modification and pain management, and 3 on suggesting supplements to participants.

**Table 3:** Probable outcomes of medication review with follow-up

Outcomes	Intervention		Control	
	Baseline	6 months	Baseline	6 months
*Frequency of medication use problems*				
Medication adminstration (median)	4.5	5	5	5
Medication adherence (median)	4.5	5	5	5
Polypharmacy (median)	4	5	4	4
Accessibility (median)	4	4	4.5	4.5
Knowledge scores (4/4)	4	4	4	4
*Drug-related outcomes, n = number of cases*				
Having more than 5 medications	0	2	4	0
Number of adverse drug events	3	5	0	0
Number of inappropriate medication case	4	2	2	0
Number of dose changes of medications	7	7	6	0
Number of total drug related problems	16	0	14	12
*Pharmacists' recommendations, n = number of suggestions*				
Medication counselling	6		2	
Suggest to the doctor to increase the dosage	4			
Suggest to the doctor to reduce the dosage	6			
Suggest to the doctor to stop inappropriate medications	3			
Suggest a change of medications	2			
Suggest supplements	3		3	
Monitoring of blood pressure and glucose readings	2		2	
Improve lifestyle modification	4		1	
Educate on proper dietary control	3			
Educate on knee joint pains remedy	1			
Suggest changing to fixed-dose combination medication	1			

#### Medication use problems frequency

This outcome highlights the feasibility of administrating the MedUseQ questionnaire to participants from both groups within the study period. Medication use problem frequency was identified through the questionnaire administration at baseline and 6 months (Table 3).

#### Drug-related problems

Finally, outcomes related to drug-related problems which were adverse drug events, number of patients with more than five medications, inappropriate medication, and dose changes were identified. The intervention group had 16 drug related problems and the control group had 14 at baseline (Table 3). At 6 months, the intervention group related issues suggested to prescribers were accepted. However, in control group, only two drug-related problems were resolved. There were statistically significant differences in the number of drug-related problems in the control group compared to the intervention group. Drug-related problems were statistically significantly higher in standard care groups (*median*= 1) than in medication review groups (*median* = 2) at 6 months, *U* = 15, *z* = − 2.98, *p* = 0.013.

## Discussion

Several studies that have been conducted on medication review in Malaysia [30, 33, 43]. To the authors’ knowledge, this study is the first pilot randomised study to assess the feasibility of conducting medication reviews with follow-up among older adults. Results demonstrate the feasibility of medication review with follow-up intervention at community pharmacies in Penang state, highlighting a signal of improvement in outcomes favouring a pharmacist-led intervention whereby (i) Medication improved medication administration, adherence and polypharmacy issues, (ii) Drug-related problems such as adverse drug events, inappropriate medications, and improper frequency and dosage were significantly lower among older adults who experienced the medication review with follow-up and (iii) Pharmacist recommendations being made to patients and prescribers. The signal of improvement in the patients receiving medication reviews from community pharmacies suggests that feasibility issues should be further considered before a definitive trial can be initiated in Malaysia.

Feasibility issues were noted by community pharmacists regarding the recruitment of participants and delivering intervention. In the recruitment phase, a longer time period was recquired to identify participants who had difficulties with medication use. Recruiting older adult participants required more time to explain and provide information on the study processes. All the community pharmacies experienced a loss of follow-up largely due to a lack of response or participants moving to another pharmacy.

Other studies have identified limitations with community pharmacies not being subsidised or integrated into the government health systems, resulting in limited transition of care, such as lack of access to medical records and follow-up [27, 44]. Currently, in Malaysia, there are no remuneration fees for services rendered in community pharmacies [7]. The lack of remuneration fees could be a barrier to the implementation of medication review as part of daily practice. Additionally, separation of prescribing and dispensing is still lacking in private settings of healthcare in Malaysia [45], which has resulted in a lack of communication between private general practitioners and community pharmacists [33]. In general, administrating the MedUseQ questionnaire was straightforward, with only one community pharmacist noting a challenge as a result of the language barrier as the questionnaire was available only in English and Malay language. Penang’s demographic mostly comprises the Chinese population, followed by the Malay and Indian population [46], thus there is a need to translate the questionnaire to other languages such as Hokkien, Mandarin and Tamil. This issue may limit initiatives for medication review in older adults [31]. Difficulities in discussing recommendations with prescribers were highlighted, largely due to lack of time from prescribers for telephone discussions. These difficulties were less in the intervention group; others have reported that general practitioners value pharmacists' skills in reviewing medications [47].

The results of our study are encouraging in terms of the identification and resolution of drug-related problems. Of note, Sellors et al. highlighted that the number of drug-related problems increases in parallel with the number of drugs and that almost 80% of older adult patients with polypharmacy have at least one drug-related problem [48]. Previous randomised controlled trials and reviews have also provided results of reducing drug-related problems and demonstrating the effectiveness of medication reviews among older adults [49, 50]. However, Toivo et al. [51] and Touchette et al. [52], reported that no effectiveness of medication review.

Similar to a small number of other studies, we focused on domains such as medication administration, medication adherence, polypharmacy, accessibility and knowledge prompting the need for medication review and deprescribing in those with complex medication regimens [53–58]. Consistent with our findings, a similar study in Malaysia reported improved medication adherence and increased knowledge score, but it is worth noting that the study populations differed.

In our study, several of the pharmacists’ recommendations were non-drug related. Recently, there has been an increasing focus on non-pharmacological approaches to promote health among older adults with recommendations such as lifestyle modification, improvement in dietary intake and integration of complementary and alternative supplements [59, 60]. Providing such recommendations is a practical approach that could improve older adults’ uptake of healthier lifestyle approaches besides correct administration of their medications and devices.

### Strengths and limitations

The main strength of this pilot randomised study is its focus on community pharmacies which could expand the provision of pharmaceutical care to older people. However, there were a few limitations which should be considered. Firstly, there was potential for recruitment bias, as the community pharmacists recruited the participants hence may have selected those more likely to have drug related problems. In addition, the community pharmacists delivered and assessed the intervention outcomes. The 6-month follow-up period may have not been sufficient to assess long term outcomes such as hospitalisation and quality of life. Furthermore, the study results may have limited generalizability, as only participants residing in Penang state were included. Of note, as a pilot randomised study, the sample sizes were not powered for the outcomes hence the statistical analysis was by defintion exploratory. 

### Implication for research and practice

Community pharmacies have a distinct advantage in delivering targeted services to older adults, enhancing their medication management. This alleviates the strain on the public healthcare system and strengthens primary care services within the country. Implementing such interventions can potentially drive evidence-based reforms applicable to both public and private primary care settings. However, future research should encompass more extensive and robust powered trials to which implement pharmacist-led interventions in community pharmacy and evaluate their impact on medication outcomes among older adults. 

## Conclusion

The results of this study demonstrate the feasibility of implementing medication review with follow-up intervention by community pharmacists and identifies potential outcomes for reducing drug-related problems, improving medication use among older adults. There is a need for definitive trials in Malaysian community pharmacy settings.

### Supplementary Information

Below is the link to the electronic supplementary material.

Supplementary file1 (DOCX 30 KB)
