# Supplementary material for: Assessing feasibility of conducting medication review with follow-up among older adults at community pharmacy: a pilot randomised controlled trial
Source: Int J Clin Pharm. 2024 Apr 18;46(4):843–53. doi: 10.1007/s11096-024-01711-3 (PMC11286689; doi:10.1007/s11096-024-01711-3)
Supplement: Supplementary file 1 — Supplementary file1 (DOCX 30 KB) [file 11096_2024_1711_MOESM1_ESM.docx]

**Supplementary materials**

**Assessing Feasibility of Conducting Medication Review with Follow-Up Among Older Adults at Community Pharmacy: A Pilot Randomized Controlled Trial**

**Contents**

Supplementary material A: Medication review according to Community pharmacy benchmarking guideline Pharmaceutical Services Division

Supplementary material B: Translated and Cross-cultural adapted Medication Use Questionnaire (MedUseQ)

Supplementary material A

Medication review according to Community pharmacy benchmarking guideline Pharmaceutical Services Division

**A) Prior to the review:**

• The pharmacist should be fully aware of the appropriate use/ dose/ administration time/ side effects and major drug interactions of each medicine the patient is taking.

• The pharmacist may need to refer to treatment guidelines as needed.

**B) During the review:**

• Welcome patient and make the patient comfortable in the consultation area.

• Explain the aims of the review.

• Explain and take the patient's consent.

• Explain that the information will be shared with their health care practitioners if needed and a record will be kept in the pharmacy.

• Confirm and complete patient details on a PM Review form.

• Record the basic health data section of the form.

• For each medicine the pharmacist should consider the following interventions:

i. Advice on medicines usage (prescribed and OTC), aiming to develop compliance;

ii. Effective use of 'when required' medicines;

iii. Ensuring appropriate use of different medicine dosage forms (e.g. inhaler and soluble tablets);

iv. Advice on possible side effects;

v. Identification of items without adequate or correct dosage instructions;

vi. Identification of unwanted medicines (if patient is no longer taking their medicines);

vii. Identification of the need for a change of dosage form to facilitate effective usage;

viii. Identification of appropriateness of medication based on Beer's criteria

ix. Proposals for dose optimization

x. Suggestions to improve patient's clinical outcome;

xi. Advice on the use of any vitamins, complementary medicines or self-purchased OTC medicines.

• Discuss the action plan with the patient and get patient's consent

• Discuss with the patient information that requires follow-up. Thank the patient and see him/her out.

**C) Post review:**

• Discuss with patient's health care practitioners if needed

• Update the patient medication record.

• File all documents systematically and securely.

• Follow up on the patient at 2 months and 4 months later

• Record number of PM Reviews performed.

Supplementary material B

# Bahagian A: Demografi

1. Umur:

_

1. Jantina:
   - Lelaki ☐Perempuan
2. Etnik:
   - Melayu ☐Cina
   - India ☐Lain-lain (Sila nyatakan)

_

1. Tahap pendidikan:
   - Tiada pendidikan formal ☐ Sekolah rendah
   - Sekolah menengah ☐Kolej/Universiti
2. Cara hidup:
   - Hidup sendiri
   - Rumah orang tua/sakit
   - Bersama keluarga
3. Apakah sumber pendapatan hidup anda?
   - Bekerja
   - Pencen
   - KWSP/ Kumpulan Wang Persaraan
   - Kebajikan sosial
   - Ahli keluarga menyara saya
4. Berapakah pendapatan isi rumah anda sebulan?
   - B40 (Kurang daripada RM4850)
   - M40 ( Rm4851-Rm10970)
   - T20 (Lebih daripada RM10971)
5. Adakah anda mempunyai insurans kesihatan?
   - Ya
   - Tidak
6. Adakah anda mempunyai kos belanja bulanan untuk kesihatan? (Termasuk perubatan dan kos rawatan kesihatan)
   - Tiada
   - Ya (Sila nyatakan dalam RM)
7. Berapa banyak ubat-ubatan semasa yang anda ambil setiap hari?

☐0

- - 1-3
  - 4-6
  - 7-10
  - Lebih daripada 10

1. Daripada siapkah anda mendapatkan bekalan ubat? (anda boleh tandakan lebih daripada satu jawapan)
   - Klinik Kesihatan Kerajaan
   - Doktor Klinik Swasta
   - Farmasi Komuniti (Kedai Farmasi)

# Bahagian B: Masalah Pengambilan Ubat

Arahan

Untuk melengkapkan soal selidik:

- Fikirkan tentang semua ubat-ubatan yang anda ambil secara tetap atau "mengikut keperluan".
- Jika soalan sesuai kepada salah satu ubat anda, jawab berdasarkan cara

pengambilan ubat tersebut.

# Jika soalan tidak sesuai dengan anda, tanda "Tidak pernah".

- Jawab setiap soalan dengan menandakan pada **satu jawapan** yang sesuai dengan anda.

# Dalam tempoh satu bulan yang lalu

- Sangat kerap = 30 kali sebulan (Setiap hari)
- Kerap = 23-29 kali sebulan
- Kadang-kadang = 9-15 kali sebulan
- Jarang = 1-8 kali sebulan
- Tidak pernah = 0 kali

Q1 Berapa kerapkah anda mempunyai masalah untuk mengambil ubat-ubatan seperti yang diarahkan kerana perubahan pada warna, saiz, atau bentuk ubat?

- Sangat kerap
- Kerap
- Kadang-kadang
- Jarang
- Tidak pernah

Q2 Berapa kerapkah anda menghadapi masalah untuk mengambil ubat kerana tidak faham apa yang diarahkan oleh doktor/farmasi?

- Sangat kerap
- Kerap
- Kadang-kadang
- Jarang
- Tidak pernah

Q3 Berapa kerapkah anda mempunyai masalah mendapatkan bekalan ubat anda tepat pada masanya? (Contoh: menunggu lama di farmasi, masalah dengan ubat pos, kesukaran berjumpa doktor untuk mendapatkan pengisian ubat semula, cuaca buruk atau tiada pengangkutan)

- Sangat kerap
- Kerap
- Kadang-kadang
- Jarang
- Tidak pernah

Q4 Berapa kerapkah anda menghadapi masalah untuk mengambil ubat seperti yang diarahkan kerana anda tidak dapat membaca atau mendengar arahan?

- Sangat kerap
- Kerap
- Kadang-kadang
- Jarang
- Tidak pernah

Q5 Berapa kerapkah anda mempunyai masalah mengambil ubat seperti yang diarahkan kerana keadaan fizikal anda menyukarkan untuk mengambilnya? (Contoh: menelan pil, membuka bekas, mengukur dos, atau menggunakan titisan mata)

- Sangat kerap
- Kerap
- Kadang-kadang
- Jarang
- Tidak pernah

Q6 Berapa kerapkah anda tidak mengambil ubat langsung atau kurang daripada yang diarahkan kerana kesan sampingan ubat tersebut menganggu aktiviti harian anda? (Contoh: perlu pergi ke bilik mandi terlalu kerap, berasa letih, atau tidak dapat berfikir dengan jelas)

- Sangat kerap
- Kerap
- Kadang-kadang
- Jarang
- Tidak pernah

Q7 Berapa kerapkah anda tidak mengambil ubat langsung atau kurang daripada yang diarahkan kerana anda cuba menjimatkan wang?

- Sangat kerap
- Kerap
- Kadang-kadang
- Jarang
- Tidak pernah

Q8 Berapa kerapkah anda tidak mengambil ubat langsung atau kurang daripada yang diarahkan kerana anda tidak suka cara pengambilan ubat tersebut? (Contoh: suntikan atau titisan mata)

- Sangat kerap
- Kerap
- Kadang-kadang
- Jarang
- Tidak pernah

Q9 Berapa kerapkah anda tidak mengambil ubat langsung atau kurang daripada yang diarahkan kerana anda fikir anda tidak memerlukannya?

- Sangat kerap
- Kerap
- Kadang-kadang
- Jarang
- Tidak pernah

Q10 Berapa kerapkah anda tidak mengambil ubat langsung atau kurang daripada yang diarahkan kerana anda fikir ubat itu tidak berkesan kepada anda?

- Sangat kerap
- Kerap
- Kadang-kadang
- Jarang
- Tidak pernah

Q11 Berapa kerapkah anda tidak mengambil ubat langsung atau kurang daripada diarahkan kerana anda bimbang akan menjadi ketagih kepada ubat- ubatan?

- Sangat kerap
- Kerap
- Kadang-kadang
- Jarang
- Tidak pernah

Q12 Berapa kerapkah anda terlupa untuk mengambil ubat?

- Sangat kerap
- Kerap
- Kadang-kadang
- Jarang
- Tidak pernah

Q13 Berapa kerapkah anda mengambil ubat tanpa preskripsi (OTC), suplemen,produk tradisional atau produk alternatif?

- Sangat kerap
- Kerap
- Kadang-kadang
- Jarang
- Tidak pernah

Q14 Berapa kerapkah anda mengambil lebih banyak ubat daripada yang telah diarahkan untuk mendapatkan lebih kelegaan daripada penyakit anda?

- Sangat kerap
- Kerap
- Kadang-kadang
- Jarang
- Tidak pernah

Q15 Berapa kerapkah anda mengambil ubat tahan sakit, ubat penenang dan ubat tidur walaupun anda memang tidak memerlukannya?

- Sangat kerap
- Kerap
- Kadang-kadang
- Jarang
- Tidak pernah

Q16 Berapa kerapkah anda perlu mengambil beberapa ubat sekaligus pada waktu yang sama?

- Sangat kerap
- Kerap
- Kadang-kadang
- Jarang
- Tidak pernah

Q17 Berapa kerapkah anda rasa anda ada terlalu banyak ubat untuk diambil?

- Sangat kerap
- Kerap
- Kadang-kadang
- Jarang
- Tidak pernah

Q18 Berapa kerapkah anda mendapati mudah untuk mendapatkan maklumat ubat anda daripada ahli kesihatan klinik dan farmasi (doktor dan farmasi)untuk rawatan kesihatan anda?

- Sangat kerap
- Kerap
- Kadang-kadang
- Jarang
- Tidak pernah

Q19 Berapa kerapkah anda pergi lebih daripada satu klinik atau farmasi komuniti (kedai farmasi) kerana anda memerlukan lebih banyak ubat daripada yang ditetapkan?

- Sangat kerap
- Kerap
- Kadang-kadang
- Jarang
- Tidak pernah

# Sila nyatakan pilihan anda

Q20 Adakah anda faham bagaimana cara menggunakan setiap ubat anda dari aspek:

| i) Masa pengambilan |  | |
| --- | --- | --- |
| - Ya | - Tidak | - Mungkin |
| ii) Dos yang betul   - Ya | - Tidak | - Mungkin |
| iii) Tujuan pengambilan   - Ya | - Tidak | - Mungkin |

iv) Kesan sampingan

- Ya ☐Tidak ☐Mungkin

# Bahagian C: Kepuasan

Arahan **Sila nyatakan tahap kepuasan anda pada soalan berikut.**

Q21 Adakah anda berpuas hati dengan perkhidmatan penggunaan ubat-ubatan yang disediakan oleh:

|  | Sangat  tidak berpuas hati | Tidak  berpuas hati | Neutral | Berpuas  hati | Sangat  berpuas hati |
| --- | --- | --- | --- | --- | --- |
| i)Farmasi Klinik Kesihatan Kerajaan | ☐ | ☐ | ☐ | ☐ | ☐ |
| ii)Klinik Swasta | ☐ | ☐ | ☐ | ☐ | ☐ |
| iii)Farmasi Komuniti (Kedai  Farmasi) | ☐ | ☐ | ☐ | ☐ | ☐ |
